# Introducing a Primary Health Care nurse training course at the University of Limpopo: Experiences and views of trainees

**DOI:** 10.4102/phcfm.v3i1.292

**Published:** 2011-11-03

**Authors:** Peter A. Delobelle, Pamela M. Mamogobo, Gert JO. Marincowitz, Rika Decock (In memoriam), AnneMarie Depoorter

**Affiliations:** 1 Department of Public Health, Vrije Universiteit Brussels, Belgium; 2 Department of Nursing, School of Health Sciences, University of Limpopo, Turfloop Campus, South Africa; 3 Department of Family Medicine and Primary Care, University of Limpopo, Polokwane Campus, South Africa

## Abstract

**Background:**

A new post-basic Primary Health Care (PHC) nurse training was piloted at the University of Limpopo in rural South Africa in order to reinforce PHC services and to address the backlog of trained PHC nurses. The training comprised residential and decentralised training modules based on the principles of problem based learning and community based education, and a patient-centred care approach developed in the field of family medicine was applied for acquiring consultation skills. Clinical reasoning was improved through on-site supervision by individual preceptors.

**Objective:**

The aim of the study was to describe the satisfaction, experiences and views of trainees in the first year of implementing the new PHC nurse training programme.

**Method:**

The study had a descriptive, exploratory and cross-sectional design, and used quantitative and qualitative methods for data collection that included a semi-structured survey questionnaire and focus group discussion. A purposive sample of trainees enrolled in the pilot programme (*n* = 15) was recruited for this study. Results were analysed quantitatively for the survey questionnaire and content analysis was used for qualitative data.

**Results:**

Results revealed trainee satisfaction with the quality of community based visits and classroom lectures and dissatisfaction with on-site supervision and training material. Qualitative findings indicated a need to improve information and communication of supervisors and preceptors, and to provide more training material. Factors related to the work environment were identified as barriers to implement learning, but the use of tools developed in family medicine curricula was perceived as beneficial. Lessons learnt included the need for strong programme coordination and stakeholder commitment, as well as the need to develop a competence framework for PHC nursing.

**Conclusion:**

The implementation of a pilot programme for PHC nurse training had the outcomes of trainee satisfaction with the mixed method of teaching, and valuable lessons were learned with regard to programme implementation and organisation. Integration of tools and concepts developed in the field of family medicine proved beneficial, and several recommendations were formulated to inform similar projects.

## Introduction

### Setting

The establishment and strengthening of Primary Health Care (PHC) services, based on the White Paper for transformation of the health system implemented through the District Health System, is a cornerstone of national health reform in South Africa.^1^ Nurses constitute the majority of health care workers in the country and form the backbone of this system, guided by a set of norms and standards articulated in the PHC Package for South Africa.^2^ The role of PHC nurses involves integrating preventive with curative services, and requires strong history-taking, diagnostic and management skills, but also effective communication and public health skills.^3^ The combination of knowledge, skills and experience needed to provide comprehensive PHC services is paramount to achieving the objective of equity in the delivery of health services, particularly in rural areas. In order to achieve this aim, a profound rethinking of curricular approaches is required, whereby nurse training needs to shift from a hospital based didactic model to a community based model.^4^

Although curricular transformation has taken place in many institutions, several studies suggest that nursing graduates continue to feel ill-prepared for practice.^5^ In addition, AIDS education is not integrated effectively in nursing curricula, which raises the question as to whether young graduates are able to cope with the increasing demand for HIV-and-AIDS services, which includes the delivery of antiretroviral treatment (ART) in PHC settings,^6^ as described in the National Strategic Plan for 2007–2011.^7^ Allowing PHC nurses to initiate and monitor people on ART, given the appropriate supervision, mentoring and support, provides greater equitable access to treatment and leads to decongestion of current ART initiation sites. Despite initiatives to improve PHC nurse competence, however, few nurses are trained for comprehensive PHC services delivery.^3^ For example, a situation analysis based on national and provincial data has estimated that about 60% of all PHC facilities lack nurses with a specific PHC training.^8^

With the objective to meet the existing PHC nurse training demand, a variety of post-basic courses is available with different options, which includes residential and decentralised learning. Formal provision of the training courses is, however, often inadequate and the responsibility for continuing education is usually left to the individual.^9^ In isolated and rural areas, such as the Limpopo Province, these programmes are not always accessible, and many nurses still lack the clinical and management skills needed to provide comprehensive PHC services.^10, 11^ In order to cope with demand and accelerate the output of trained nurses, a new training programme was hence developed at the University of Limpopo, Campus Turfloop, through a series of consultations with academic and government stakeholders. The programme comprises a 1-year PHC nurse training that includes residential and decentralised training modules and which is accessible to professional nurses with a work experience of at least 2 years.

The curriculum is outcomes and competency based in line with current recommendations,^10^ and was developed in partnership with the Department of Family Medicine and PHC, using innovative tools for developing clinical and management skills and a patient-centred care (PCC) approach for learning consultation skills. The programme covers specific aspects of PHC nursing, including the provision of HIV-and-AIDS care, and is based on the principles of problem based learning (PBL) and community based education (CBE). The residential part includes classroom lectures and clinical practice in local clinics, which are alternated with in-service training modules under supervision of trained preceptors. This study was designed to explore the barriers and facilitators to implementing the mixed residential and decentralised learning programme, and to share the lessons learnt.

### Objectives

The aim of this study was to describe the satisfaction, experiences and views of trainees in the pilot year of implementing the new PHC nurse training programme.

## Ethical considerations

The study was approved by the Provincial Department of Health Research Ethics Committee as part of a large-scale research project involving the University of Limpopo and the Vrije Universiteit Brussel, Belgium. Informed consent was obtained orally from all participants; confidentiality was ensured, and results were discussed with students in a plenary group session.

## Methods

This study had a descriptive, exploratory and cross-sectional design, and utilised both quantitative and qualitative methods for data collection. The study was conducted during a classroom session that was organised as part of the residential programme of the PHC training course. A purposive sample was used, which included all students enrolled in the first year of implementation of the new training course (*n* = 18), of whom those present at the time of data collection were included in this study (*n* = 15).

Data was collected with a semi-structured questionnaire which comprised a scale and open-ended questions related to satisfaction with training aspects, such as the quality of classroom lectures and community visits, on-site training and supervision, training material, workload, and research assignments. The questionnaire was scored along a 5-point Likert scale, with answers ranging from ‘Very dissatisfied’ (1), through ‘Neither satisfied nor dissatisfied’ (3), to ‘Very satisfied’ (5), with higher scores indicating more satisfaction. The open-ended questions included, ‘What would you advise in order to improve the next module of the PHC training?’, and ‘What would you advise in order to improve on-site training and supervision?’ In addition, students needed to report if they had been supervised by their preceptor or mentor in the month before evaluation; whether the supervision and training resources supported their work as PHC nurses; and whether the case studies of the training were representative of the clinical setting.

A focus group discussion was organised as well, where trainees were asked to reflect on the enabling factors and barriers to the implementation of their learning as PHC nurses, and to offer comments and suggestions for improving on-site training and supervision. The focus group discussion was led by the main study researcher and facilitated by the PHC nurse training coordinator who utilised a structured interview guide. Notes were taken and main findings were discussed in plenary after the focus group discussion using an overview grid.

Scale results were analysed quantitatively with SPSS version 15.0, whereby mean satisfaction scores were calculated for individual scale items. Data from the open-ended questions, notes and transcripts from the discussion were analysed through qualitative content analysis, which facilitated the production of core constructs from textual data and the generation of contextual meaning.^12^ A conventional approach was used, which included open coding, abstraction, and creating categories through inductive analysis.^13^ Credibility of analysis was improved through seeking peer agreement on the production of categories and coding, and by discussing themes with participants in a plenary group session.^14^

## Results

**FIGURE 1:**
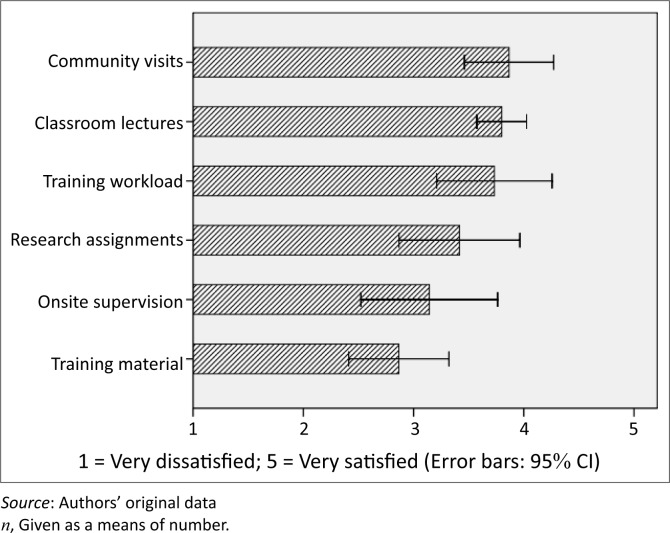
Mean satisfaction with selected Primary Health Care training aspects (*n* = 15).

Results of the evaluation showed an overall student satisfaction with the PHC training (M = 3.5; s.d. = 0.4). Students were (Figure 1) satisfied the most with the quality of community visits (M = 3.9; s.d. = 0.7) and classroom lectures (M = 3.8; s.d. = 0.4), and satisfied the least with training material (M = 2.9; s.d. = 0.8), which mostly comprised hand-outs and manuals, and on-site supervision (M = 3.1; s.d. = 1.1). The training workload was considered satisfactory (M = 3.7; s.d. = 1.0), and research assignments neither satisfactory nor unsatisfactory (M = 3.4; s.d. = 0.9).

Three in four nurses reported that they had been supervised by their mentor or preceptor in the month preceding the assessment (72%). Nearly all of the respondents (93%) who had received supervision agreed that this enabled them to perform their duties as PHC nurses more efficiently, and half (50%) reported that the supervision was used to discuss problems. Less than half (44%) reported that their clinical work had been physically observed, or that performance was discussed, and only four in ten reported that clinical issues were discussed (38%). Some nurses mentioned that they had been supervised by persons other than their mentor or preceptor, such as immediate supervisors (28%) or hospital doctors (22%), in the preceding month.

All but one nurse reported that the training material assisted them in their work and that the case studies reflected the type of patients encountered in daily practice (94%). One in five of the trainees (22%), however, felt that their work environment did not enable them to practice as PHC nurses, because of staff shortages or supervisors who were unaware of the training.

These results were confirmed by qualitative findings, which highlighted several barriers and facilitators to the implementation of learning. Major themes concerned the need for improved information and communication, training support, and conditions related to the work environment (Table 1).

**TABLE 1:** Content analysis of focus group transcript and open-ended questions (*n* = 15).

Theme	Category	Subcategory
Information, communication	Colleagues, supervisors	Awareness of training programme
		Recognition of student status
Training & support	On-site supervision	Frequency of supervision
		Number of trained preceptors
	Training material	Referenced textbooks
Work environment	Resource availability	Counselling space
		Equipment
		Staff shortages

*Source*: Authors’ original data *n*, Given as a means of number.

The relay of information to and communication with supervisors and peers, as well as respect for the PHC student status, was found to be crucial in enabling PHC nurses to apply their newly acquired knowledge, as illustrated by the following quote: ‘Please contact our mentors and inform them about the programme and the course outline; ensure that our student status is respected and acknowledged by other health care workers’.

Many participants requested that supervisors and preceptors be informed adequately or trained in the PHC training format and that more supervision was conducted. The participants asked for more time and support to apply the newly acquired knowledge and suggested some short test of answering questions, to be conducted during training to establish whether they understood the learned material. They also demanded more information and training material in order to support the process of individual learning, as demonstrated by the following: ‘We need hand-outs and textbooks, if possible, to look up relevant information; please provide us with some library references in order to consult after the end of the lecture’.

With regard to the tools used for practice support, however, most participants agreed that this enabled them to improve their decision-making skills, and they asked that other PHC nurses be trained as well. Participants also requested that all preceptors are trained in the application of the training tools through a training-of-the-trainer seminar or a so-called *crash course*. Participants were also satisfied with the visit of a local HIV-and-AIDS clinic, which one nurse described as ‘a real eye-opener’, in learning how to cope effectively with the additional workload imposed by HIV and AIDS.

Nonetheless, issues related to the work environment such as lack of equipment and counselling space as well as chronic staff shortages, were perceived by some as hindering the implementation of the new training format, as illustrated by the following: ‘Please consider full-time study leave in order for us not to replace staffing shortages’.

## Discussion

This study presents the experiences and views of trainees enrolled in the pilot implementation of a new PHC nurse training course which includes both residential and decentralised training modules. The programme provides key demonstrations and exposure to the PHC setting, and focusses on the development of consultation skills and the effective management of common illnesses and health related problems by using the principles of problem based learning (PBL) and community based education (CBE).

PBL can be defined as an approach toward learning and instruction in which students tackle problems in small facilitated group settings, based on the philosophy of a process-oriented curriculum in which knowledge is actively constructed rather than acquired, and in which teaching staff is committed to guiding rather than transmitting knowledge.^15^ In this respect, PBL is centred around the acquisition of critical reflexive thinking skills, and has been identified as a method of choice in PHC teaching.^10^ In this study, trainees were generally satisfied with this method of training, but they recommended the use of more training material to assist with the individual learning process.

In CBE, the community, especially in under-developed and under-resourced settings, is used extensively for learning purposes to ensure the relevance of education to local population needs.^16^ As an educational philosophy, CBE aims to equip professionals with problem solving skills and positive attitudes towards society, which, in turn, are grounded in the essence of equality as witnessed in a democratic society.^17^ CBE has also been identified as the best way to prepare nurses for integration in the new district based health-care system in South Africa.^18^

In this study, trainees were most satisfied with community based visits, but least satisfied with onsite supervision, which was attributed mainly to a lack of information about, and communication with, preceptors. Preceptors are experienced registered nurses who provide support and guidance to student nurses during clinical placement, which has proven to be an efficient educational strategy for community nursing in South Africa.^19^ In-service training has the advantage of increasing the relevance of training to the local context, whilst at the same time saving time and travelling expenses, and developing awareness of the programme amongst other staff members.^20^

The use of residential and in-service training modules, however, presents several challenges in terms of administrative and logistical organisation. Individual follow-ups and community visits of rural PHC clinics require a strong and lasting commitment from both the faculty and the preceptors, which was reflected by comments from students in regard to the supervision received during training. Mixed method programmes also require a committed counterpart at government level to drive the process and to allocate the necessary resources, which may range from designing job descriptions for preceptors to granting study leave to students. Stakeholder commitment should be continuous as well, to ensure smooth implementation and follow-up of clinical and research activities. The use of individual preceptors hence requires strong programme coordination to provide adequate and timely information and motivation. Lack of transparency in this regard can lead easily to frustration and can eventually affect the quality of PHC services.

The results confirm what is known about implementing CBE in nursing education in South Africa, indicating the need for collaborative efforts and true partnerships between academic institutions and communities, supported by government involvement.^21^ Shifting nurse education from the traditional classroom-based approach to CBE creates distinctive problems and demands more from facilitators, which requires a continuous support of the faculty.^22^ Sustained programme evaluation and adaptation of support staff is needed thus to achieve desired outcomes and ultimately reflects on the quality of services delivery. This study formed part of such an evaluation and resulted in learning several valuable lessons.

Integration of tools and methods developed in the field of family medicine offered a clear benefit, because the knowledge developed elsewhere was implemented without duplicating efforts. The integration of HIV-and-AIDS treatment and care in the training modules was also appreciated by trainees and should be made mandatory in all nursing curricula. Some useful models have been developed already in health sciences curricula, and have proven their success.^23^ In effect, nurse leaders from educational institutions in South Africa have recently agreed to implement a standardised HIV-and-AIDS-related curriculum.^24^

Finally, in order to improve learning skills and to avoid confusion related to expected programme outcomes, learning objectives should be developed. The formulation of learning objectives reinforces the competence base of nurse training and needs to be based on the requirements of a competence framework for PHC nurses, which has recently been developed in South Africa for reasons of benchmarking.^3^ This seems all the more important, given the need to delineate the scope of practice of PHC nurses related to the envisaged deployment of mid-level health workers at district level in the near future.

### Limitations of the study

This study was based on the views and experiences of a small sample of PHC trainees and is hence indicative of the described programme and period of time. Although the study was limited to describing the satisfaction of learners, such an evaluation forms an essential first step in the sequence of curriculum evaluation, which typically consists of four levels of evaluation.^25^ In this pilot, evaluation was limited to the assessment of trainee satisfaction with PHC nurse training because of time and financial constraints. This is, however, considered important because, (1) it provides valuable feedback which helps to evaluate the programme, (2) it reassures students that their input is needed to judge programme effectiveness, (3) it provides quantitative information, and (4) it can be used to define performance standards.^25^

In addition, a survey analysis was conducted using an investigator-developed questionnaire without established validity. Survey results were, however, triangulated with qualitative findings and found to be congruent, thereby confirming its validity. The aim of triangulation is to provide completeness and confirmation, which increases validity and reliability by improving the trustworthiness of data as well as assisting in the interpretation of data.^26^

## Conclusion

This study has indicated satisfaction amongst the trainees of a new 1-year postgraduate PHC training programme, guided by the principles of CBE and PBL, whilst adopting a patient-centred approach. The use of residential and decentralised training modules presented several challenges, which required sustained stakeholder commitment and strong programme coordination in order to meet expectations about individual follow-up and supervision. The integration of tools for learning clinical and management skills, developed in the field of family medicine, was also beneficial, in particular with regard to HIV-and-AIDS treatment and care.

The programme allowed PHC nurses to practice in their own environment supervised by trained preceptors, and contributes to strengthening PHC services delivery in the public health sector, in which investment in training and follow-up of individual nurses and preceptors translates into enhanced quality of care for entire communities. Consequently, it is advisable to explore the possibilities of widening the implementation of this and similar training models to other areas and health service providers, pending the available government and administrative support.

## References

[1] Department of Health White paper for the transformation of the health system in South Africa. Pretoria: National Department of Health; 1997.

[2] Department of Health The primary health care package for South Africa: a set of norms and standards. Pretoria: Department of Health; 2000.

[3] Strasser S, London L, Kortenbout E. Developing a competence framework and evaluation tool for primary care nursing in South Africa. Educ Health (Abingdon) [serial online]. 2005 [cited Dec 17 2010];18(2). Available from: http://search.ebscohost.com/login.aspx?direct=true&db=afh&AN=17575573&site=ehost-live 10.1080/13576280500145615 16009609

[4] Strachan K, Clarke E. The Nursing Curriculum: How is it developed? HST Update. Durban: Health Systems Trust; 2000 p. 10–12.

[5] Lehmann U. Strengthening human resources for primary health care In: Barron P, Roma-Reardon J, editors. South African Health Review 2008. Durban: Health Systems Trust, 2008; p. 163–178.

[6] Breier M, Wildschut A, Mgqolozana T. Nursing in a new era: The profession and education of nurses in South Africa. Cape Town: HSRC Press; 2009.

[7] Department of Health HIV & AIDS and STI Strategic Plan for South Africa, 2007–2011. Pretoria: Department of Health; 2007.

[8] Department of Health Strategic Priorities for the National Health System, 2004–2009. Pretoria: Department of Health; 2004.

[9] Woods D, Cope F, Eley B. The challenge of providing HIV training to health professionals. South Afr J HIV Med. 2008;9(3):15–17.

[10] Mekwa J. Transformation in nursing education In: Ntuli A, editor. South African Health Review 2000. Durban: Health Systems Trust, 2000; p. 271–283.

[11] Mabaso SS. Evaluation of a decentralised primary health care training programme [unpublished MA Dissertation]. University of South Africa; 2006.

[12] Priest H, Roberts P, Woods L. An overview of three different approaches to the interpretation of qualitative data. Part 1: theoretical issues. Nurse Researcher. 2002;10(1):30–42. PMid:12405004 12405004 10.7748/nr2002.10.10.1.30.c5877

[13] Elo S, Kyngas H. The qualitative content analysis process. J Adv Nurs. 2008;62(1):107–115. http://dx.doi.org/10.1111/j.1365-2648.2007.04569.x, PMid:18352969 18352969 10.1111/j.1365-2648.2007.04569.x

[14] Graneheim UH, Lundman B. Qualitative content analysis in nursing research: concepts, procedures and measures to achieve trustworthiness. Nurse Educ Today. 2004;24(2):105–112. http://dx.doi.org/10.1016/j.nedt.2003.10.001, PMid:14769454 14769454 10.1016/j.nedt.2003.10.001

[15] Uys LR, Gwele NS. Curriculum development in nursing: Process and innovation. New York: Routledge; 2005 http://dx.doi.org/10.4324/9780203313343

[16] Mtshali NG. Conceptualisation of community-based basic nursing education in South Africa: a grounded theory analysis. Curationis. 2005;28(2):5–12. 16045106 10.4102/curationis.v28i2.939

[17] Villani CJ, Atkins D. Community-Based Education. Sch Community J. 2001;11(1):121–126.

[18] Radebe G. Nurse training for the District Health System. HST Update. 2000;54:5–6.

[19] Setswe G. Clinical accompaniment in community nursing. Nurs Stand. 2002;16(45):33–36. 12219417 10.7748/ns2002.07.16.45.33.c3236

[20] Strasser S, Gwele N. Nurse Oriented Primary Health Care In: Ntuli A, editor. South African Health Review 1998. Durban: Health Systems Trust, 1998; p. 83–92.

[21] Mtshali NG. Implementing community-based education in basic nursing education programs in South Africa. Curationis. 2009;32(1):25–32. PMid:20225750 20225750 10.4102/curationis.v32i1.870

[22] Fichardt AE, Du Rand PP. Facilitators’ perceptions of problem-based learning and community-based education. Health SA Gesondheid. 2000;5(2):3–10.

[23] McLean M, Hiles L. Introducing HIV and aids education into the first year of a problem-based learning curriculum: a template for health science education. Health SA Gesondheid. 2008;10(2):17–23.

[24] Dohrn J, Nzama B, Murrman M. The Impact of HIV Scale-Up on the Role of Nurses in South Africa: Time for a New Approach. J Acquir Immune Defic Syndr. 2009;52:S27–S29. http://dx.doi.org/10.1097/QAI.0b013e3181bbc9e4, PMid:19858933 10.1097/QAI.0b013e3181bbc9e4 19858933

[25] Kirkpatrick DL, Kirkpatrick JD. Evaluating training programs: The four levels. 3rd ed San Fransisco, CA: Berrett-Koehler; 2006.

[26] Halcomb EJ, Andrew S. Triangulation as a method for contemporary nursing research. Nurse Researcher. 2005;13(2):71–82. PMid:16416981 27707301 10.7748/nr.13.2.71.s8

